# Minimizing Oxidative Stress in Oral Surgery: A Comparative Study of Laser-Assisted and Conventional Third Molar Extractions

**DOI:** 10.3390/dj12120402

**Published:** 2024-12-10

**Authors:** Paul Șerban Popa, Elisabeta Claudia Popa-Cazacu, Anamaria Zaharescu, Gabriel Valeriu Popa, Mădălina Nicoleta Matei

**Affiliations:** Faculty of Medicine and Pharmacy, ”Dunărea de Jos” University of Galați, 47 Domnească Str., 800181 Galați, Romania; paul.popa@ugal.ro (P.Ș.P.); cc445@student.ugal.ro (E.C.P.-C.); anamaria.zaharescu@ugal.ro (A.Z.); madalina.matei@ugal.ro (M.N.M.)

**Keywords:** salivary biomarkers, oxidative stress, third molar extraction, laser-assisted surgery, total antioxidant capacity (TAC), wound healing

## Abstract

**Background/Objectives:** This study aims to compare the effects of conventional surgical techniques and laser-assisted methods on salivary oxidative stress biomarkers following third molar extraction, in order to evaluate the potential benefits of laser surgery in reducing oxidative stress and promoting faster recovery. **Methods:** A total of 154 patients, aged 16–30, undergoing third molar extractions were included in the study. Patients were divided into two groups: conventional surgery (*n* = 75) and laser-assisted surgery (*n* = 79). Saliva samples were collected at baseline, and 24, 48, 72, and 168 h postoperatively. The levels of total antioxidant capacity (TAC), malondialdehyde (MDA), and 8-hydroxy-2′-deoxyguanosine (8-OHdG) were measured as indicators of oxidative stress. **Results:** Initial biomarker levels were similar across all participants. Postoperative oxidative stress increased in both groups, with significantly higher levels in the conventional surgery group at 48 and 72 h. Salivary biomarkers of oxidative stress were significantly lower in the laser group at 48 and 72 h post-surgery (*p* < 0.05), indicating a faster recovery. By 168 h, biomarker levels in the laser group had nearly returned to baseline, whereas levels in the conventional group remained slightly elevated. **Conclusions:** Laser-assisted surgery significantly reduces oxidative stress and promotes faster recovery when compared with conventional methods, as evidenced by the more rapid normalization of salivary biomarkers. These findings suggest that laser techniques may offer superior clinical outcomes in third molar extractions.

## 1. Introduction

### 1.1. Saliva as a Diagnostic Medium

In recent years, saliva has gained considerable attention as a valuable, non-invasive diagnostic tool, reshaping both clinical research and practice. Unlike traditional diagnostic methods that often depend on blood sampling, saliva provides a much more patient-friendly alternative [1]. Collecting saliva is not only simple and painless but also cost-effective, making it an appealing option for monitoring various physiological and pathological states. The wide range of biological markers present in saliva—including enzymes, hormones, antibodies, and oxidative stress biomarkers—makes it a versatile medium that can reflect both local and systemic health conditions [2]. The growing interest in saliva as a diagnostic tool is driven by its potential for real-time monitoring, offering valuable insights into an individual’s health without the need for invasive procedures. Numerous studies have demonstrated its usefulness in diagnosing and monitoring a range of diseases, including oral cancer, cardiovascular conditions, diabetes, and infectious diseases, among others [3]. The non-invasive nature of saliva collection, along with its capacity to reflect systemic conditions, highlights its growing role in personalized medicine. By enabling continuous, less burdensome health monitoring, saliva, as a diagnostic tool, holds the potential to revolutionize patient care [4]. As a non-invasive medium, saliva allows for the real-time monitoring of oxidative stress, which is crucial in wound healing and postoperative recovery. This makes it especially valuable for assessing how different surgical techniques impact the recovery process.

### 1.2. The Importance of Salivary Oxidative Stress

Oxidative stress is a key factor in the development of many diseases, making its detection and monitoring essential in both research and clinical settings. It occurs when there is an imbalance between the production of reactive oxygen species (ROS) and the body’s antioxidant defenses, leading to cellular and molecular damage. This imbalance plays a central role in the onset and progression of numerous conditions, including cancer, neurodegenerative disorders, and cardiovascular diseases [5]. Salivary oxidative stress specifically reflects the oxidative status within the oral cavity and can act as a useful marker for systemic oxidative stress levels. This is especially important in oral health, where local oxidative stress can drive the onset and progression of oral diseases, while also providing insights into broader systemic health issues. By measuring salivary biomarkers related to oxidative stress—such as total antioxidant capacity (TAC), malondialdehyde (MDA), and 8-hydroxy-2′-deoxyguanosine (8-OHdG)—researchers can gather valuable information about the body’s overall oxidative balance [6]. The importance of salivary oxidative stress goes beyond its role in disease development. It is increasingly recognized as a key factor in understanding the body’s response to various stressors, including surgical procedures. For example, elevated oxidative stress levels have been associated with delayed wound healing and a higher risk of postoperative complications, making it a critical consideration when evaluating surgical outcomes [5,7]. Therefore, exploring salivary oxidative stress in different clinical scenarios can provide valuable insights into the interplay between oxidative damage and the body’s antioxidant defenses.

### 1.3. Overview of Salivary Oxidative Biomarkers

Studying oxidative stress in saliva relies on the precise measurement of specific biomarkers that reflect the body’s oxidative status. Of these, total antioxidant capacity (TAC), malondialdehyde (MDA), and 8-hydroxy-2′-deoxyguanosine (8-OHdG) are the most extensively researched and clinically relevant [8].

Total antioxidant capacity (TAC): TAC represents the combined activity of all antioxidants present in saliva, offering a comprehensive view of the body’s ability to neutralize reactive oxygen species (ROS). It serves as a crucial marker in oxidative stress studies because it provides a broad overview of the antioxidant defenses available to counteract oxidative damage. Measuring TAC in saliva helps researchers evaluate the overall oxidative balance and assess the potential risk of oxidative stress-related damage [9,10].

Malondialdehyde (MDA): MDA is a byproduct of lipid peroxidation, a process in which free radicals attack lipids in cell membranes, leading to cellular damage. Elevated MDA levels in saliva indicate heightened oxidative stress and are commonly used as markers of cellular damage. By monitoring MDA levels, researchers can gain insights into the extent of lipid peroxidation happening in the body, making it a valuable biomarker in the study of oxidative stress-related conditions [9,10].

8-hydroxy-2′-deoxyguanosine (8-OHdG): 8-OHdG is a biomarker that indicates oxidative damage to DNA, resulting from ROS attacks. This damage can lead to mutations and contribute to carcinogenesis, underscoring the importance of 8-OHdG as a marker for genomic instability. Measuring 8-OHdG levels in saliva provides a non-invasive way to assess the impact of oxidative stress on DNA integrity, which is particularly relevant in studying cancer and other diseases driven by genetic mutations. Given the role oxidative stress plays in tissue damage and healing, salivary biomarkers like these offer valuable tools for evaluating the body’s response to surgical interventions, such as third molar extractions [9,10].

### 1.4. The Relevance of Third Molar Pathologies

The third molars, or wisdom teeth, are often associated with various pathologies, making them a frequent subject of study in oral and maxillofacial research. Common issues related to third molars include impaction, pericoronitis, cyst formation, and the resorption of adjacent teeth [11]. Among these, impaction is the most prevalent, occurring when the tooth fails to fully erupt into the oral cavity. Impacted third molars can lead to a range of complications, including inflammation, pain, and infection, which often necessitate surgical intervention [12]. Surgical extraction of impacted third molars is one of the most common procedures in oral surgery, yet it is not without risks. The complexity of the extraction, particularly in cases of impaction, can lead to significant postoperative discomfort and complications [13]. These include prolonged healing times, infection, and in some cases, permanent nerve damage. Given the frequency of these complications, understanding the factors that influence postoperative outcomes is crucial for improving patient care.

Wound healing following third molar extraction is a complex process that unfolds in several stages: hemostasis, inflammation, proliferation, and remodeling. Each stage plays a vital role in ensuring the successful repair of the extraction site and the restoration of normal tissue architecture [14].

Hemostasis: This initial phase occurs immediately after the tooth is removed and involves the formation of a blood clot to stop bleeding and protect the wound from external contaminants.

Inflammation: During this phase, immune cells are recruited to the site of injury to clear debris and prevent infection. The inflammatory response is crucial for setting the stage for tissue repair, but excessive or prolonged inflammation can impede healing and increase the risk of complications.

Proliferation: The proliferation phase involves the formation of new tissue, including granulation tissue and the re-epithelialization of the wound surface. This stage is characterized by the rapid production of collagen and other extracellular matrix components that provide structural support for the regenerating tissue.

Remodeling: The final phase of wound healing is marked by the maturation of collagen fibers and the restoration of the normal architecture of the tissue. The remodeling phase can take several weeks to months, depending on the individual and the complexity of the extraction.

The efficiency and speed of wound healing following third molar extraction can be influenced by a variety of factors, including the surgical technique used, the patient’s overall health, and the presence of systemic conditions that may impair the healing process. Understanding these factors is essential for optimizing surgical outcomes and minimizing the risk of postoperative complications [15,16].

### 1.5. Research Question and Study Significance

The growing interest in laser-assisted surgical techniques for third molar extractions stems from their potential to minimize postoperative complications, reduce tissue trauma, and promote faster wound healing when compared with conventional methods. Conventional surgery often results in significant postoperative oxidative stress due to the tissue damage caused by the mechanical trauma and thermal effects from drilling. Oxidative stress, characterized by an imbalance between reactive oxygen species (ROS) production and the body’s antioxidant defenses, can impede healing and contribute to delayed recovery. Previous studies have shown that oxidative stress biomarkers, such as total antioxidant capacity (TAC), malondialdehyde (MDA), and 8-hydroxy-2′-deoxyguanosine (8-OHdG), are useful indicators of the extent of tissue damage and the recovery trajectory in postoperative patients [1,4,5].

Laser-assisted techniques, on the other hand, are hypothesized to reduce oxidative stress by promoting precise tissue incision with minimal collateral damage and through photobiomodulation, which has been shown to enhance cellular repair processes and reduce inflammation [17]. Furthermore, studies in related surgical fields have reported accelerated healing and reduced postoperative complications with the use of lasers, suggesting that they may also modulate oxidative stress responses [18,19,20].

Based on the existing literature, we hypothesize that laser-assisted third molar extraction will result in significantly lower levels of oxidative stress biomarkers, specifically TAC, MDA, and 8-OHdG, at 24, 48, and 72 h post-surgery compared with conventional extraction methods. We further hypothesize that these lower levels of oxidative stress will correlate with faster recovery, evidenced by the earlier return of these biomarkers to baseline levels in patients undergoing laser-assisted surgery. This hypothesis is grounded in the assumption that the reduced thermal and mechanical tissue trauma inherent in laser surgery will lead to diminished ROS production, thereby facilitating a more rapid resolution of oxidative stress and faster tissue regeneration.

The primary objective of this study is to quantify and compare the changes in salivary oxidative stress biomarkers (TAC, MDA, 8-OHdG) between conventional and laser-assisted third molar extractions. By capturing postoperative biomarker levels at multiple time points (24, 48, 72, and 168 h), we aim to determine whether laser surgery can significantly attenuate oxidative stress and promote more efficient wound healing compared with traditional surgical methods. Secondary objectives include assessing the relationship between oxidative stress biomarkers and clinical outcomes such as pain, swelling, and patient-reported recovery times.

If our hypothesis is confirmed, the findings could provide compelling evidence to support the adoption of laser-assisted surgical techniques in clinical practice for third molar extractions. Reduced oxidative stress and faster recovery times would represent substantial benefits for patients, potentially leading to fewer complications, shorter recovery periods, and improved overall outcomes. Additionally, the study may contribute to the growing body of literature on the role of oxidative stress in postoperative healing and the therapeutic advantages of laser technologies in oral surgery.

## 2. Materials and Methods

This study compares two surgical methods for third molar extraction: the conventional surgical technique and a laser-assisted surgical technique, both of which are commonly employed in oral and maxillofacial surgery.

Conventional surgical method: The conventional technique for third molar extraction involves making an incision in the gum tissue using a scalpel, followed by the removal of any overlying bone with a dental drill if necessary, and the extraction of the tooth with forceps. While this method is widely used, it is associated with significant tissue trauma, which can result in considerable postoperative pain, swelling, and extended healing times [21]. The tissue trauma induced by the scalpel and drill often leads to a prolonged inflammatory response, which can delay the overall wound healing process and increase patient discomfort during recovery [22].

Laser surgical method: In contrast, the laser-assisted surgical technique utilizes a dental laser to perform the incision and tooth removal. The laser provides several advantages over conventional methods, including greater precision, reduced bleeding, and minimized tissue damage [18]. Specifically, the laser promotes faster coagulation, which reduces the risk of postoperative bleeding and bacterial contamination. The use of lasers also results in less thermal damage to surrounding tissues, which is a common concern with drills and scalpel use [23]. This method is believed to facilitate quicker healing and reduce postoperative discomfort, contributing to improved patient outcomes. The laser parameters used in this study were as follows: the LX16 Plus Diode Laser, provided by Guilin Woodpecker Medical Instrument Co. Ltd., Guangxi, P.R. China, was operated at wavelengths of 450 ± 20 nm (maximum power of 3 W) and 976 ± 20 nm (maximum power of 5 W). The power was applied with a spot size of approximately 2–3 mm, resulting in a calculated fluence of 23.9 to 10.6 J/cm^2^ for the 450 nm wavelength and 39.8 to 17.7 J/cm^2^ for the 976 nm wavelength, depending on the spot size. The laser was used for a mean time of 3.47 min during the procedure for each patient.

Beside the patient-related factors, surgery-specific variables, such as the degree of difficulty of the extraction, duration of the surgery, and intraoperative tissue trauma, may confound the relationship between the surgical method and oxidative stress responses. Longer or more complex surgeries are likely to result in increased tissue damage and a higher inflammatory response, thus elevating oxidative stress biomarkers [17,20]. To mitigate this, the complexity and duration of each surgery were standardized as much as possible, with all procedures performed by experienced surgeons following the same operative protocol. Additionally, the degree of impaction and surgical complexity were recorded for each patient and included as covariates in the statistical analysis to adjust for any potential confounding effects related to these factors. This approach allowed us to isolate the effects of the surgical technique on postoperative oxidative stress from those attributable to surgical difficulty.

In both the conventional and laser-assisted surgery groups, platelet-rich fibrin (PRF) was utilized to promote healing. PRF was prepared using a two-step centrifugation process to concentrate platelets from the patient’s own blood, following established protocols for autologous PRF preparation as described by Mahanani et al. (2010) [24]. PRF product was applied directly to the surgical site following extraction, in order to leverage its high concentration of growth factors and thus facilitate tissue regeneration and reduce inflammation. The use of PRF has been shown to improve wound healing in oral surgeries by promoting angiogenesis and minimizing local oxidative stress [25]. In our study, PRF was used consistently in both surgical groups to ensure standardization across procedures and to mitigate the potential for differential healing.

Platelet-rich fibrin (PRF) membranes were employed in both the conventional and laser-assisted surgical procedures to enhance wound healing. PRF, a second-generation platelet concentrate, is obtained by centrifuging the patient’s blood without the use of anticoagulants. This process results in a fibrin matrix that is rich in platelets, leukocytes, and growth factors, all of which play critical roles in tissue regeneration and repair. The PRF membranes were prepared using a clinical centrifuge (model CF2415, Dr. Mayer Ltd., Bucharest, Romania) that operates at speeds ranging from 1000 to 4000 rpm, with a maximum centrifugal force of 1933 rcf. The centrifugation process for PRF was standardized at 3000 rpm for 10 min, yielding a dense fibrin network that was subsequently applied to the surgical site. The inclusion of PRF in the surgical protocol is intended to accelerate the healing process by promoting angiogenesis, enhancing epithelialization, and reducing inflammation. The use of PRF has been shown to significantly improve healing outcomes, particularly in oral surgery settings such as third molar extractions [26].

Saliva samples were collected from each patient in order to evaluate oxidative stress biomarkers before and after the third molar extraction. The collection method involved allowing unstimulated saliva to gather at the floor of the mouth, from where it was collected every 60 s using transfer pipettes until a total volume of 5 mL was reached. The samples were then mixed thoroughly using a vortex mixer and divided into 1 mL portions in separate Eppendorf tubes. Each tube was centrifuged at 1500× *g* for 2 min, and the resulting supernatant was collected and stored at −80 °C to maintain biomarker integrity and prevent degradation [27]. Saliva samples were collected at five critical time points: preoperatively (baseline), and at 24, 48, 72, and 168 h postoperatively. This timeline was selected in order to capture both the acute and subacute changes in oxidative stress biomarkers, providing a comprehensive view of the healing process and the differential effects of the surgical methods used [28,29].

To ensure consistency in the surgical procedures and postoperative outcomes, the following inclusion and exclusion criteria were applied to the study participants:

Inclusion criteria:

- Impacted or submucosal third molars: Only patients with impacted or submucosal third molars were included to maintain uniformity in the surgical approach and to ensure comparable postoperative conditions across the study population.
- Good oral hygiene: Participants were required to have good oral hygiene, as assessed by a simplified oral hygiene index (OHI-S) score of less than 1.2. This was necessary to reduce the risk of postoperative infection and to ensure that healing outcomes were not confounded by poor oral health [30].
- Age and gender: The study included both male and female participants aged 16 to 30 years, a demographic commonly undergoing third molar extractions.

Exclusion criteria:

- Systemic conditions: Patients with systemic conditions such as diabetes, leukemia, herpes, cancer, or oral candidiasis were excluded in order to avoid potential confounding factors that could influence oxidative stress levels or healing outcomes [31].
- Medications and treatments: Patients who had received antibiotics or fluoride treatment within the two weeks prior to surgery were excluded to prevent any potential interference with the study’s biomarkers.
- Other factors: Smokers and women currently menstruating were also excluded, as these factors are known to affect oxidative stress levels and could introduce variability into the study results [32].

The oxidative stress biomarkers analyzed in this study included total antioxidant capacity (TAC), malondialdehyde (MDA), and 8-hydroxy-2′-deoxyguanosine (8-OHdG), each of which was assessed using specific assay kits.

Total antioxidant capacity (TAC): The TAC of saliva was measured using a colorimetric assay kit (ab65329, Abcam Limited, Cambridge, UK) designed for the sensitive and accurate quantification of antioxidant proteins and small molecules in biological samples. The assay involved the preparation of a copper ion reagent, which was added to the saliva samples and standards in a 96-well microplate. After incubation, the absorbance was measured at 570 nm using a microplate reader. The TAC levels were determined by comparing the absorbance of the samples to a standard curve which was generated using Trolox, a water-soluble vitamin E analog.

Malondialdehyde (MDA): MDA, a biomarker of lipid peroxidation, was quantified using the Lipid Peroxidation (MDA) Assay Kit (ab118970, Abcam Limited, Cambridge, UK), which measures MDA levels through the formation of an MDA–thiobarbituric acid (MDA–TBA) adduct that can be detected colorimetrically at 532 nm. Saliva samples were first lysed using MDA Lysis Buffer, and the MDA–TBA adduct was generated by incubating the samples with TBA at 35 °C for 60 min. The resulting mixture was then cooled and the absorbance was measured to determine the MDA concentration.

8-hydroxy-2′-deoxyguanosine (8-OHdG): The oxidative DNA damage marker 8-OHdG was measured using an ELISA kit (ab285254, Abcam Limited, Cambridge, UK), which allows for the quantitative determination of 8-OHdG levels in saliva. The assay protocol involved the dilution of saliva samples and standards, which were added to a microplate pre-coated with an anti-8-OHdG antibody. Following the addition of a biotinylated detection antibody and HRP-streptavidin conjugate, the signal was developed using TMB substrate and measured at 450 nm. The concentration of 8-OHdG was calculated based on a standard curve.

The study’s hypotheses were structured to evaluate the impact of surgical methods on salivary oxidative stress biomarkers. The null hypothesis (H_0_) posited that there would be no significant difference in the levels of these biomarkers—total antioxidant capacity (TAC), malondialdehyde (MDA), and 8-hydroxy-2′-deoxyguanosine (8-OHdG)—between patients undergoing third molar extraction via conventional surgical methods and those treated with laser-assisted surgery at any postoperative time point. In contrast, the alternate hypothesis (H_a_) suggested that significant differences would be observed, specifically predicting lower levels of these biomarkers in the laser-assisted group at one or more postoperative time points. These hypotheses guided the statistical analysis, aiming to determine whether the choice of surgical technique influenced oxidative stress levels in saliva.

The study employed a cohort design, which involved the prospective observation of two distinct groups of patients undergoing third molar extraction, either through conventional surgery or laser-assisted surgery. This design was chosen to allow for the direct comparison of postoperative outcomes, particularly focusing on the levels of oxidative stress biomarkers in saliva at various time points following surgery.

A power analysis was conducted to determine the minimum sample size required to detect a statistically significant difference in salivary oxidative stress biomarkers (TAC, MDA, and 8-OHdG) between the conventional and laser-assisted surgery groups. Based on data from previous studies on oxidative stress biomarkers in surgical patients [5,13,18,20], we expected a moderate effect size (Cohen’s d = 0.5). An alpha level (α) of 0.05 was set to control for the risk of a Type I error, and the study was powered at 80% (1 − β = 0.8) in order to detect significant differences between the groups. Using these parameters, the power analysis indicated that a minimum of 64 participants per group (128 total) would be needed to detect a significant effect at 24, 48, and 72 h post-surgery. To ensure robustness and to account for potential dropouts or incomplete data, we aimed to recruit a total of 154 participants (75 in the conventional surgery group and 79 in the laser-assisted surgery group), which exceeds the minimum requirement and provides adequate power for detecting clinically significant differences between the two groups. We successfully recruited all targeted participants, resulting in a total of 154 patients, aged 16 to 30, undergoing third molar extractions. These patients were allocated into two groups: 75 in the conventional surgery group and 79 in the laser-assisted surgery group. The calculated minimum sample size was increased by approximately 20% to account for potential participant dropout, incomplete data, or deviations during the study.

Participants were recruited from the university hospital’s oral surgery department, and all eligible patients meeting the inclusion criteria were invited to participate. Upon enrollment, patients were randomly assigned to either the conventional surgery group or the laser-assisted surgery group. Randomization was achieved using a computer-generated random sequence, ensuring that the allocation was concealed from both the participants and the surgical team until the time of surgery. This method of randomization helps to minimize selection bias and ensures that any differences observed between the groups can be attributed to the intervention rather than confounding variables.

To further enhance the study’s rigor, a double-blind approach was adopted. The patients were blinded to the type of surgical procedure they received, as were the laboratory personnel analyzing the saliva samples. This blinding was crucial in preventing bias in patient-reported outcomes and in the measurement of biomarker levels. The surgeons performing the procedures were not blinded due to the nature of the interventions, but they were not involved in the postoperative care or in the outcome assessment, thereby reducing the potential for bias.

All statistical analyses were conducted using Stata/BE 18 software, a powerful tool widely used for biostatistics and epidemiological studies. Initially, descriptive statistics were generated for all variables, including patient demographics, baseline biomarker levels, and clinical outcomes. Means, standard deviations, medians, and interquartile ranges were calculated for continuous variables, while frequencies and percentages were reported for categorical variables. These statistics provided an overview of the study population and ensured that the randomization process had produced comparable groups at baseline.

The primary analysis focused on comparing the levels of oxidative stress biomarkers (TAC, MDA, and 8-OHdG) between the two surgical groups at each postoperative time point. For the statistical analysis, paired sample *t*-tests were used to assess within-group changes in biomarker levels from baseline to each subsequent time point. Independent sample *t*-tests were used to compare differences in biomarker levels between the two surgical groups. These tests were selected due to their ability to accurately detect differences between two groups, particularly for within-group and between-group comparisons in a two-group study design. This approach enhances the precision of the analysis and aligns with the study design, which involves comparing two groups across various time points.

A power analysis was conducted using Stata’s power calculation tools to determine the appropriate sample size needed to detect a clinically significant difference in biomarker levels between the two groups. The power analysis was based on prior studies, with an alpha level of 0.05 and a power of 0.8, ensuring that the study was adequately powered to detect differences that were both statistically and clinically significant.

The normality of the data was assessed using the Shapiro–Wilk test prior to conducting any statistical analysis. Depending on the outcome of the normality test, the appropriate measures of central tendency and variability were selected, as follows: mean and standard deviation (SD) for normally distributed data and median and interquartile range (IQR) for non-normally distributed data.

Sensitivity analyses were performed to assess the robustness of the study findings. This involved re-running the analyses under different assumptions, such as varying the imputation method for missing data or excluding outliers. The results of the sensitivity analyses were consistent with the main findings, providing additional confidence in the study’s conclusions.

One potential source of bias in this study is the inherent variability in patients’ baseline oxidative stress levels, which could influence postoperative measurements. Factors such as lifestyle, diet, overall health, and pre-existing conditions (e.g., inflammation or systemic diseases) could introduce significant variability in the levels of oxidative stress biomarkers (TAC, MDA, 8-OHdG) prior to surgery. These individual differences, particularly in antioxidant capacity, could affect the magnitude of postoperative oxidative stress responses, potentially confounding the comparison between conventional and laser-assisted surgical techniques. To mitigate this bias, baseline saliva samples were collected from all participants prior to surgery and used as a reference point for postoperative comparisons. By normalizing postoperative biomarker levels to each patient’s baseline, we aim to account for inter-individual variability. Additionally, patients were screened for and excluded if they had systemic conditions known to influence oxidative stress (e.g., diabetes, cardiovascular diseases, chronic inflammatory conditions).

Dietary habits, particularly the intake of antioxidants (e.g., vitamins C and E, or polyphenols), are known to influence the body’s oxidative status. Moreover, factors such as smoking, alcohol consumption, and stress levels can significantly elevate ROS production and impair the body’s antioxidant defenses. Variability in these factors among participants may obscure the true effects of the surgical interventions on oxidative stress levels. While controlling for all lifestyle factors is challenging in a clinical setting, we minimized their impact by excluding smokers and patients with a history of heavy alcohol consumption from the study. Furthermore, all participants were instructed to refrain from using antioxidant supplements or making significant dietary changes during the study period. Although not all lifestyle factors could be controlled, their potential impact was acknowledged, and future studies could include dietary assessments to further refine this analysis.

The use of pain medications, anti-inflammatory drugs, or other treatments can alter oxidative stress levels, either by directly influencing ROS production or by modulating the body’s inflammatory response [18]. For example, nonsteroidal anti-inflammatory drugs (NSAIDs), commonly used postoperatively, have antioxidant properties and may confound the measurement of oxidative stress biomarkers. To address this, postoperative pain was managed with standardized pain relief protocols that avoided medications with significant antioxidant effects. This approach helped minimize the influence of external treatments on biomarker levels, ensuring that differences observed between the surgical groups could more confidently be attributed to the surgical techniques themselves.

The study received ethical approval from the University Ethics Committee (Comisia de Etică Universitară—CEU) under the University “Dunărea de Jos” of Galați. The approval was granted following a thorough review of the study protocol, which included an assessment of the potential risks and benefits to participants, as well as the measures taken to protect participant confidentiality and data integrity. The CEU’s approval was documented in H_CEU no. 20 from 3 July 2024, which confirmed that the study complied with all relevant ethical guidelines and regulations.

Informed consent was obtained from all participants before their inclusion in the study. The consent process was conducted in accordance with Romanian law (Law 95/2006 regarding healthcare reform and Law 46/2003 on patient rights) and international ethical guidelines, including the Declaration of Helsinki and the CIOMS guidelines. Participants were provided with a comprehensive explanation of the study’s purpose, procedures, potential risks, and benefits through an informed consent document. This document detailed the nature of the surgical interventions, the collection and use of biological samples, and the study’s objectives. For participants under the age of 18, informed consent was obtained from a parent or legal guardian. The consent process ensured that participants (or their guardians) fully understood that participation was voluntary and that they could withdraw from the study at any time without any impact on their medical care. The consent form also clarified that participants would not receive any direct benefits from the study, nor would they be informed of the individual results of the research. However, the knowledge gained from the study could potentially benefit future patients by improving understanding of oxidative stress in surgical recovery.

Strict confidentiality measures were implemented to protect participants’ personal data. Upon enrollment, each participant was assigned a unique identification code that was used to label all biological samples and data records. This coding system ensured that individual identities were protected throughout the study and in any subsequent publications. All data, including saliva samples and clinical records, were stored securely and were accessible only to authorized personnel involved in the study. In compliance with European Union data protection regulations (GDPR), participants were informed that their anonymized data might be shared with international research databases. The informed consent document clearly outlined the procedures for data handling and the steps taken to ensure that personal information remained confidential. Moreover, the consent form specified that any future use of the samples or data for additional research would require further ethical approval, ensuring ongoing oversight and protection of participants’ rights. The consent document explicitly stated that any future use of the samples would adhere to strict ethical guidelines and would be subject to further review to ensure that participants’ rights and confidentiality were maintained.

## 3. Results

### 3.1. Descriptive Statistics and Baseline Biomarker Levels

Descriptive statistics were calculated for key study variables, including patient demographics, baseline levels of oxidative stress biomarkers (TAC, MDA, and 8-OHdG), and clinical outcomes. Continuous variables, such as biomarker concentrations and age, were summarized using means, standard deviations, medians, and interquartile ranges (IQRs). Categorical variables, such as gender and surgical method (conventional or laser-assisted), were expressed as frequencies and percentages. These statistics provided an overview of the baseline comparability between groups and ensured the normality of data before proceeding with further statistical analysis. The sample size of 154 participants was adequate to detect significant differences in oxidative stress biomarkers between the conventional and laser-assisted groups. This sample size was sufficient for detecting moderate differences, contributing to the reliability of the findings.

Before surgery, the levels of oxidative stress biomarkers were consistent across participants, indicating no significant differences in baseline oxidative status between groups. TAC values ranged from 0.2 to 1.5 mmol/L, MDA levels from 0.2 to 0.8 μmol/L, and 8-OHdG levels from 0.5 to 5.0 ng/mL, all within the expected physiological ranges. This uniformity in baseline biomarker levels validates the subsequent comparisons between conventional and laser-assisted surgical methods. Normality tests (Shapiro–Wilk) were performed to determine the distribution of the baseline oxidative stress biomarker data. TAC and MDA levels were found to be normally distributed, whereas 8-OHdG levels exhibited a non-normal distribution. Therefore, baseline data for TAC and MDA are presented as mean ± SD, and 8-OHdG data are presented as median (IQR). See Table 1.

### 3.2. Postoperative Biomarker Changes

#### 3.2.1. 24 h Post-Surgery: Biomarker Changes

At 24 h post-surgery, all oxidative stress biomarkers—total antioxidant capacity (TAC), malondialdehyde (MDA), and 8-hydroxy-2′-deoxyguanosine (8-OHdG)—increased in both surgical groups, indicating an initial oxidative stress response due to the surgical trauma. Paired sample *t*-tests were conducted for within-group comparisons, revealing significant increases from baseline for both groups (*p* < 0.05). However, the independent sample *t*-tests showed no significant differences between the conventional and laser-assisted groups at this early time point (*p* > 0.05), suggesting similar initial oxidative stress responses. The Shapiro–Wilk test indicated that the oxidative stress biomarkers TAC and MDA remained normally distributed, whereas 8-OHdG continued to show non-normal distribution at 24 h post-surgery. Therefore, TAC and MDA are presented as mean ± SD, while 8-OHdG is presented as median (IQR). See Table 2.

#### 3.2.2. 48 h Post-Surgery: Biomarker Changes

At 48 h post-surgery, the paired sample *t*-tests indicated continued increases in oxidative stress biomarkers for both groups compared with baseline (*p* < 0.05). However, the independent sample *t*-tests revealed significant differences between the two groups: the laser-assisted group had significantly lower levels of TAC, MDA, and 8-OHdG compared with the conventional surgery group (*p* < 0.01). These findings suggest that laser-assisted surgery mitigates oxidative stress more effectively during the recovery process. At 48 h post-surgery, normality testing showed that TAC and MDA remained normally distributed, while 8-OHdG continued to be non-normally distributed. Consequently, TAC and MDA values are reported as mean ± SD, and 8-OHdG as median (IQR). See Table 3.

#### 3.2.3. 72 h Post-Surgery: Biomarker Changes

By 72 h post-surgery, paired sample *t*-tests showed a significant reduction in biomarker levels for the laser-assisted group compared with earlier time points (*p* < 0.01), while levels in the conventional surgery group remained elevated. Independent sample *t*-tests confirmed significant differences between the two groups (*p* < 0.01), with the laser-assisted group demonstrating faster recovery and significantly lower oxidative stress biomarker levels than the conventional group. These results further highlight the advantages of laser-assisted surgery in accelerating the reduction of oxidative stress. Normality tests indicated normal distribution for TAC and MDA but non-normal distribution for 8-OHdG at 72 h post-surgery. Therefore, TAC and MDA are presented as mean ± SD, and 8-OHdG as median (IQR). See Table 4.

#### 3.2.4. 168 h Post-Surgery: Biomarker Changes

At 168 h (7 days) post-surgery, paired sample *t*-tests showed that the laser-assisted group’s biomarker levels had nearly returned to baseline, while the conventional surgery group continued to display slightly elevated levels of TAC, MDA, and 8-OHdG. Independent sample *t*-tests revealed significant differences between the two groups (*p* < 0.05), with the laser-assisted group experiencing significantly lower oxidative stress levels. These findings indicate that laser-assisted surgery is associated with a more rapid resolution of oxidative stress compared with conventional surgery. The Shapiro–Wilk test showed that TAC and MDA returned to normal distribution, while 8-OHdG remained non-normally distributed at 168 h post-surgery. TAC and MDA values are thus presented as mean ± SD, and 8-OHdG as median (IQR). See Table 5.

**Figure 1:**
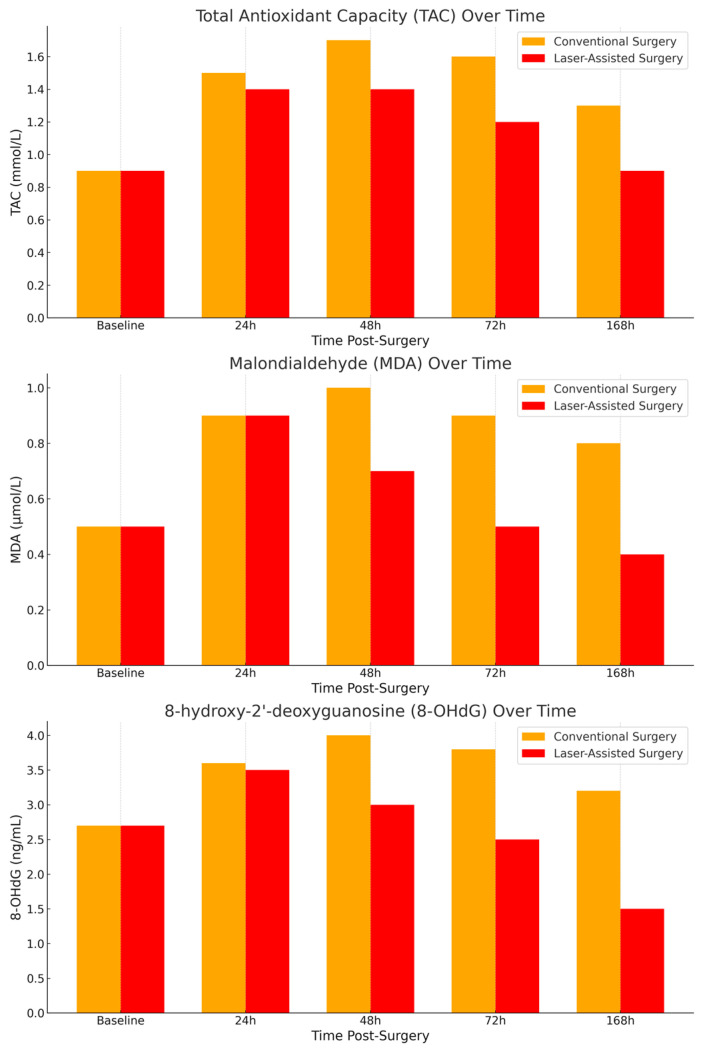
Biomarker levels over time.

**Figure 2:**
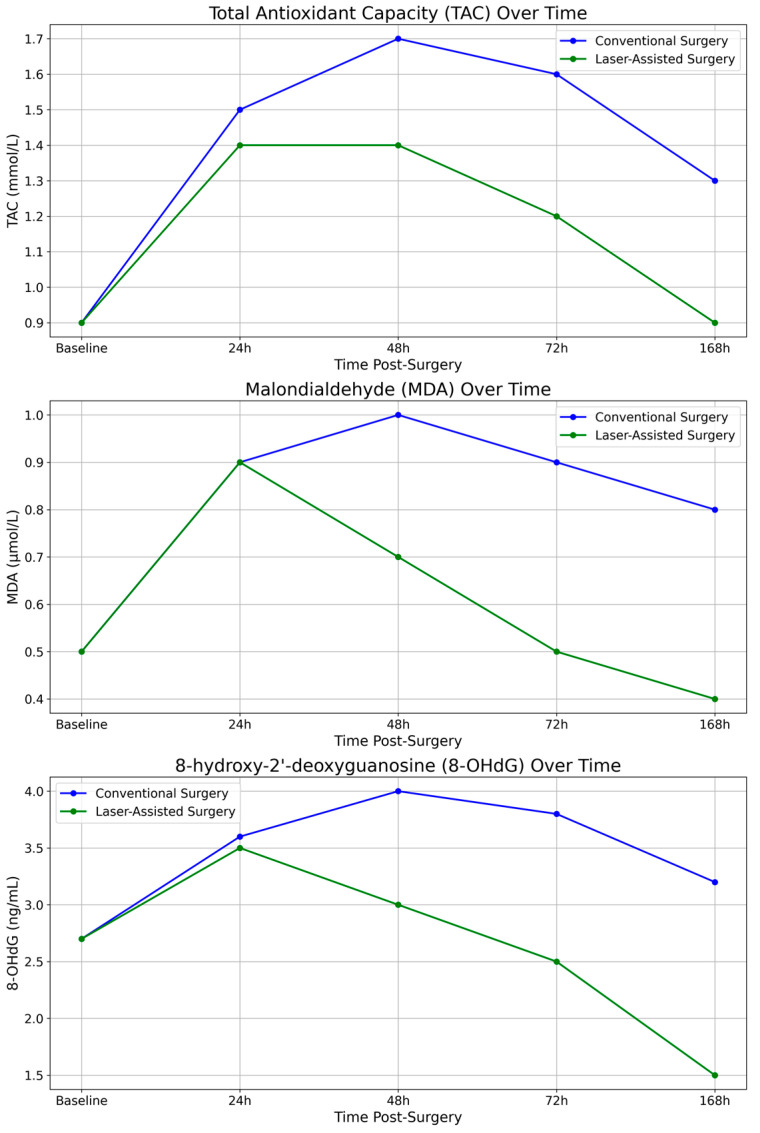
Biomarker trends over time.

**Figure 3:**
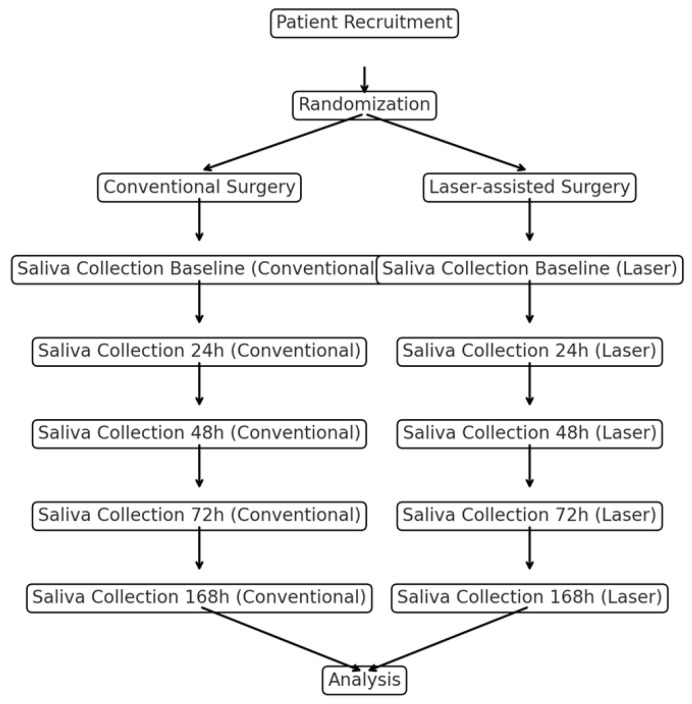
Study’s flowchart.

To further elucidate the differences in postoperative oxidative stress responses between the two surgical techniques, we measured the salivary levels of total antioxidant capacity (TAC), malondialdehyde (MDA), and 8-hydroxy-2′-deoxyguanosine (8-OHdG) at multiple time points (24 h, 48 h, 72 h, and 168 h post-surgery). The bar graphs in Figure 1 provide a clear comparison of the mean values for each biomarker across the two groups (conventional vs. laser-assisted surgery) at each time point. Meanwhile, the line graphs in Figure 2 illustrate the trends in oxidative stress biomarker levels over time, highlighting the recovery trajectories in each group. Both graphical representations underscore the significantly lower oxidative stress levels in the laser-assisted group, with more rapid normalization of biomarkers compared with the conventional surgery group. These findings suggest that laser-assisted surgery may facilitate faster tissue recovery and mitigate oxidative damage more effectively. A detailed flowchart (Figure 3) depicting the study design is provided to illustrate the overall process, from patient recruitment and randomization to postoperative saliva collection and biomarker analysis. This schematic representation outlines the flow of participants through each stage of the study, highlighting the standardized protocols followed for both the conventional and laser-assisted surgery groups. It also details the time points at which salivary samples were collected and analyzed, allowing for a clear understanding of the study’s methodology and timeline.

## 4. Discussion

The current study aimed to compare laser-assisted and conventional third molar extraction techniques, focusing on their impact on salivary oxidative stress biomarkers—total antioxidant capacity (TAC), malondialdehyde (MDA), and 8-hydroxy-2′-deoxyguanosine (8-OHdG). The findings provide evidence that laser-assisted surgery offers several advantages over conventional methods, particularly in terms of faster healing and reduced postoperative oxidative stress. This discussion explores the implications of these findings, their alignment with existing literature, and potential limitations and future research directions.

The results of this study suggest that laser-assisted surgery promotes faster healing compared with conventional methods, as evidenced by the quicker reduction in oxidative stress biomarkers in the laser group, particularly at 48 and 72 h postoperatively. The significant decrease in MDA and 8-OHdG levels at these time points suggests reduced lipid peroxidation and oxidative DNA damage, which are markers of tissue injury. These findings align with previous studies that have shown lasers to induce less tissue trauma due to their precision and ability to seal blood vessels, leading to less postoperative inflammation and swelling [20]. In particular, the ability of lasers to minimize oxidative stress might be explained by their photobiomodulation properties, which stimulate cellular repair and enhance mitochondrial function, thereby promoting tissue regeneration [33]. This is consistent with the quicker resolution of TAC levels in the laser group, suggesting that antioxidant defenses recover more swiftly following laser surgery, reducing the duration of oxidative stress. These findings reinforce the hypothesis that laser-assisted techniques not only reduce tissue damage during surgery but also enhance the body’s ability to recover from surgical trauma more efficiently.

One of the key findings in this study is the improved patient comfort reported by individuals undergoing laser surgery. This observation can be attributed to multiple factors, including reduced tissue damage and the inherent analgesic properties of lasers, which are well documented in the literature [19]. Lasers minimize nerve damage by sealing nerve endings during surgery, which reduces postoperative pain and swelling. Furthermore, the anti-inflammatory effects of laser therapy may also contribute to better pain control and reduced discomfort during the recovery period [34]. These findings support the growing body of evidence suggesting that laser-assisted surgeries can enhance the patient experience, making them a more favorable option for clinical practice, especially in cases where postoperative comfort is a priority.

The study found that salivary oxidative stress levels, as measured by TAC, MDA, and 8-OHdG, were significantly lower in the laser group compared with the conventional group, particularly at 48 and 72 h post-surgery. This reduction in oxidative stress may reflect a more efficient wound healing process and a lower inflammatory response in the laser-assisted group. The rapid decline in oxidative stress biomarkers in the laser group is consistent with previous studies that have demonstrated the ability of lasers to reduce oxidative stress markers and promote faster recovery [35]. Oxidative stress is known to play a critical role in delayed wound healing and postoperative complications. The lower levels of oxidative stress biomarkers observed in the laser group at 168 h post-surgery further support the hypothesis that laser-assisted surgery reduces the burden of oxidative stress, leading to a more favorable healing trajectory. This finding is particularly important because prolonged oxidative stress can interfere with tissue regeneration and contribute to complications such as infection or delayed wound closure [36].

Our findings are consistent with previous studies that have evaluated the effects of laser-assisted surgery on postoperative healing and oxidative stress. For example, Zhang et al. (2020) investigated the impact of diode laser-assisted periodontal surgery on oxidative stress markers, demonstrating a significant reduction in MDA levels and enhanced total antioxidant capacity (TAC) compared with conventional surgery [20]. Their study, like ours, suggests that laser technology minimizes tissue damage and associated inflammatory responses, leading to a reduction in reactive oxygen species (ROS) production. The mechanisms underlying this effect are thought to include photobiomodulation, which enhances cellular repair processes, reduces pro-inflammatory cytokine release, and promotes angiogenesis in the surgical site [19].

Additionally, our findings align with those of Buranasin et al. (2023), who reported a significant decrease in oxidative stress markers, including 8-OHdG, following laser-assisted maxillofacial surgeries [5]. Their study demonstrated that laser-assisted techniques facilitate tissue healing by minimizing mechanical trauma and thermal injury. As observed in our study, patients in the laser-assisted group had lower oxidative stress levels at early postoperative time points, suggesting more rapid recovery compared with the conventional surgery group. These results further support the role of laser surgery in reducing surgery-induced oxidative stress.

The application of diode lasers in oral surgery has been extensively studied, with particular attention to parameters such as wavelength, power density, and fluence, which significantly influence clinical outcomes. For instance, a scoping review by Parker (2013) highlights that diode lasers with wavelengths ranging from 810 to 980 nm are associated with reduced pain, minimal bleeding, and expedited recovery in soft tissue procedures [37]. This aligns well with our findings, where the use of the 976 nm diode laser facilitated effective tissue ablation with favorable postoperative healing. Furthermore, the study by Romanos et al. (2023) on the efficacy of five laser wavelengths (450, 532, 808, 1064, and 1340 nm) demonstrated significant postoperative benefits, including reduced inflammation and enhanced wound healing [38]. The outcomes observed in our study with 450 nm and 976 nm wavelengths are consistent with those reported, indicating the appropriateness of our selected laser parameters. Similarly, Gupta et al. (2020) examined the effectiveness of low-level diode laser therapy in enhancing wound healing after gingivectomy, emphasizing the importance of selecting the correct laser parameters for achieving optimal clinical results [39]. This is supported by our study, which observed reduced oxidative stress markers and quicker recovery with laser-assisted surgery when compared with conventional methods. In addition, Sharma et al. (2024) investigated the impact of a 970 nm infrared diode laser on the regenerative potential of human periodontal ligament stem cells, discussing the importance of fluence and power settings [40]. This finding aligns with our results, where the fluence values used (ranging from 10.6 to 39.8 J/cm^2^) appeared optimal for minimizing thermal damage and promoting healing. Lastly, Akbulut et al. (2013) evaluated the effectiveness of the 810 nm diode laser in oral soft tissue therapy and provided a comparative analysis with other wavelengths, demonstrating that such lasers provide effective results with minimal adverse effects [33]. This supports our conclusion that the 976 nm laser used in our study provided effective results with reduced postoperative complications.

The clinical relevance of reducing oxidative stress has been underscored in other studies. For example, Kazakova (2023) found that patients undergoing diode laser-assisted surgeries had fewer postoperative complications, such as pain and swelling, compared with those undergoing conventional surgery [41]. This observation may explain our findings of reduced oxidative stress levels, as oxidative stress has been closely linked to prolonged inflammation and delayed healing in surgical wounds [42].

Our results also corroborate the findings of Ferrante et al. (2023), who studied the effect of laser-assisted third molar surgery on oxidative stress biomarkers and postoperative pain [39]. Their study demonstrated that patients undergoing laser-assisted surgery had significantly lower MDA and TAC levels postoperatively, consistent with our findings. Importantly, their study highlighted a correlation between reduced oxidative stress and improved clinical outcomes, such as decreased pain and faster tissue regeneration, further emphasizing the clinical benefits of laser technology.

Despite the promising results, there are several potential biases and confounding factors that should be considered when interpreting the findings of this study. One key limitation is the inherent variability in patients’ baseline oxidative stress levels. Factors such as individual differences in oxidative stress due to lifestyle, diet, or underlying health conditions may have influenced the results. López-Jornet et al. (2024) and Maló et al. (2016) have highlighted that lifestyle factors like diet and stress can significantly impact oxidative stress biomarkers, which is consistent with the variability observed in our study [43,44]. While the study attempted to control for some confounders, such as excluding smokers and patients with systemic diseases, other factors, like patients’ use of pain medications or stress levels, were not controlled, which could have influenced biomarker levels postoperatively. The study’s design also poses limitations. Although the randomized design reduces selection bias, the relatively small sample size, and the exclusion of certain patient populations (e.g., smokers and individuals with chronic conditions) limit the generalizability of the findings. Buranasin et al. (2023) have reported that including diverse populations in laser-assisted surgical studies is critical for assessing broad applicability, which underscores the limitations of our current exclusion criteria [5]. Furthermore, the study did not measure the long-term outcomes of laser-assisted surgery, and the follow-up period of seven days may not be sufficient to capture the full extent of healing differences between the two methods. Future studies with larger, more diverse populations and extended follow-up periods are needed to validate these findings and explore the long-term implications of laser-assisted surgery.

The application of PRF in both the conventional and laser-assisted surgery groups warrants careful consideration, as it may have influenced the observed reduction in oxidative stress markers and enhanced healing. PRF is well-documented for its role in accelerating tissue repair through the release of growth factors, such as platelet-derived growth factor (PDGF) and transforming growth factor-beta (TGF-β), which promote angiogenesis and tissue regeneration [45]. Studies have demonstrated that PRF can significantly reduce local inflammation and oxidative stress, thereby contributing to improved postoperative outcomes [46]. In the context of this study, PRF was used across both groups to standardize postoperative care, ensuring that the observed differences were attributable to the surgical techniques rather than variations in healing treatment; however, it also introduces a potential confounding factor, as it may have masked some of the differences attributable solely to the surgical techniques employed. Future research should consider either eliminating PRF or comparing outcomes with and without PRF application to fully elucidate its role in the observed effects.

The study’s exclusion criteria (e.g., excluding patients with chronic diseases and smokers) inherently limit the generalizability of the findings, particularly to populations with coexisting health conditions or differing baseline oxidative stress profiles. This introduces potential bias, as the study’s results may not be applicable to broader clinical populations that would typically present for third molar extraction. Zhang et al. (2024) have highlighted similar limitations in their study of laser-assisted periodontal therapy, emphasizing that the exclusion of certain populations can affect external validity [20]. While we acknowledge that the strict exclusion criteria were necessary to maintain a homogeneous study population and minimize confounding variables, this also limits the external validity of the study. Future studies should consider including more diverse populations, such as those with systemic conditions, to assess whether the findings hold true across different patient demographics and health statuses.

The findings from this study suggest that laser-assisted third molar extractions offer several clinical benefits, including faster recovery, reduced oxidative stress, and better patient comfort. These advantages have important implications for clinical practice, particularly in cases where rapid healing and minimal postoperative discomfort are crucial. For example, patients who require third molar extractions due to infections or other complications could benefit from laser-assisted techniques, which may reduce the risk of postoperative complications and improve overall patient outcomes. Ferrante et al. (2013) found that laser-assisted surgery led to decreased postoperative pain and faster tissue regeneration, which aligns with our findings of reduced oxidative stress in the laser group [39]. Additionally, the reduced oxidative stress observed in the laser group points to the potential for fewer postoperative complications, such as delayed healing or infection. Given the growing body of evidence supporting the use of lasers in oral surgery, clinicians may consider adopting laser-assisted techniques more widely, particularly in complex cases where minimizing tissue damage and oxidative stress is critical for successful outcomes.

One strength of this study is the robust sample size, which was determined through a power analysis based on previous studies evaluating oxidative stress biomarkers in surgical settings. Our final sample of 154 participants exceeded the minimum required sample size (128 participants) to detect moderate differences between the conventional and laser-assisted surgery groups. This ensures that the study was adequately powered to identify statistically and clinically significant changes in biomarker levels, minimizing the risk of Type II errors. However, despite the adequate sample size, some variability in biomarker responses due to individual differences in baseline oxidative stress levels may have affected the precision of our findings. Although the sample size was adequate for the detection of moderate differences in the primary outcomes, it may limit the broader generalizability of the findings, particularly when considering smaller effect sizes or subgroup analyses. The exclusion of certain populations, such as smokers and individuals with chronic conditions, may also reduce the diversity of the study population. Future research with larger, more heterogeneous populations is warranted to explore the broader applicability of laser-assisted surgery in third molar extractions across different patient demographics.

Furthermore, while the sample size calculation was based on estimates derived from similar studies, the power of the study to detect smaller effect sizes may be limited. Han et al. (2023) noted that larger sample sizes in laser-assisted surgical trials are crucial for detecting subtle but clinically significant differences in outcomes, which may be an area for future research [36]. Future studies with larger, more diverse populations could explore more subtle differences in biomarker responses, potentially revealing additional benefits of laser-assisted surgery in patients with varying baseline oxidative stress levels.

While this study provides valuable insights into the benefits of laser-assisted surgery, several avenues for future research remain. First, studies with larger and more diverse populations are needed to confirm the findings and ensure that they are generalizable to a broader patient base. Additionally, future research should explore the long-term outcomes of laser-assisted third molar extractions, particularly in relation to postoperative complications, recurrence of symptoms, and patient satisfaction. Moreover, mechanistic studies that investigate the specific pathways by which lasers reduce oxidative stress and promote healing could provide a deeper understanding of the therapeutic effects of laser surgery. Such research could lead to the development of new surgical protocols that optimize the use of laser technology to further improve patient outcomes. Finally, comparative studies evaluating the cost-effectiveness of laser-assisted surgery compared with conventional methods would be valuable, particularly considering the potential for reduced recovery times and complications. Romanos (2021) has suggested that cost-effectiveness analyses are key to assessing the broader adoption of laser technologies in clinical settings [19]. Uncontrolled factors such as variations in patients’ dietary intake of antioxidants or differences in immune response could have influenced the biomarker levels observed. Future studies should consider dietary standardization or tracking of additional lifestyle variables to enhance the accuracy of biomarker assessments.

While we took significant measures to minimize bias and control for confounding variables, we acknowledge that some residual confounding may still exist due to uncontrollable patient or procedural factors. Nevertheless, by employing baseline normalization, exclusion criteria, standardized surgical protocols, and statistical adjustments, we have significantly reduced the risk of bias and strengthened the validity of our findings.

## 5. Conclusions

In conclusion, the results of this study highlight the potential benefits of laser-assisted third molar extractions, particularly in terms of reducing oxidative stress, promoting faster healing, and enhancing patient comfort. While the findings are promising, further research is needed to address the study’s limitations and explore the long-term implications of laser surgery. Nonetheless, the reduced oxidative stress and improved healing outcomes observed in this study suggest that laser-assisted surgery may be a superior option for third molar extractions, warranting consideration in clinical practice.

## Figures and Tables

**Table 1:** Descriptive statistics of baseline biomarkers and patient demographics.

Variable	Mean ± SD/Median (IQR)	Range	Frequency (%) for Categorical Variables
TAC (mmol/L)	0.9 ± 0.3	0.2–1.5	-
MDA (μmol/L)	0.5 ± 0.2	0.2–0.8	-
8-OHdG (ng/mL)	2.6 (1.9–3.5)	0.5–5.0	-
Age (years)	23 (21–26)	16–30	-
Gender (male/female)	-	-	82 (53%)/72 (47%)
Surgical method	-	-	78 (51%) conventional 76 (49%) laser-assisted

**Table 2:** Biomarker levels at 24 h post-surgery.

Biomarker	Mean ± SD (Conventional)	Median (IQR) (Conventional)	Mean ± SD (Laser)	Median (IQR) (Laser)	*p*-Value
TAC (mmol/L)	1.5 ± 0.4	-	1.4 ± 0.3	-	0.62
MDA (μmol/L)	0.9 ± 0.3	-	0.9 ± 0.2	-	0.68
8-OHdG (ng/mL)	-	3.5 (2.9–4.3)	-	3.4 (2.8–4.1)	0.73

**Table 3:** Biomarker levels at 48 h post-surgery.

Biomarker	Mean ± SD (Conventional)	Median (IQR) (Conventional)	Mean ± SD (Laser)	Median (IQR) (Laser)	*p*-Value
TAC (mmol/L)	1.7 ± 0.5	-	1.4 ± 0.3	-	0.01
MDA (μmol/L)	1.0 ± 0.3	-	0.7 ± 0.2	-	0.01
8-OHdG (ng/mL)	-	4.0 (3.2–4.8)	-	3.0 (2.5–3.5)	0.01

**Table 4:** Biomarker levels at 72 h post-surgery.

Biomarker	Mean ± SD (Conventional)	Median (IQR) (Conventional)	Mean ± SD (Laser)	Median (IQR) (Laser)	*p*-Value
TAC (mmol/L)	1.6 ± 0.4	-	1.2 ± 0.3	-	0.01
MDA (μmol/L)	0.9 ± 0.3	-	0.5 ± 0.2	-	0.01
8-OHdG (ng/mL)	-	3.8 (3.0–4.5)	-	2.5 (2.0–3.0)	0.01

**Table 5:** Biomarker levels at 168 h post-surgery.

Biomarker	Mean ± SD (Conventional)	Median (IQR) (Conventional)	Mean ± SD (Laser)	Median (IQR) (Laser)	*p*-Value
TAC (mmol/L)	1.3 ± 0.3	-	0.9 ± 0.2	-	0.01
MDA (μmol/L)	0.8 ± 0.2	-	0.4 ± 0.1	-	0.01
8-OHdG (ng/mL)	-	3.2 (2.6–3.8)	-	1.5 (1.1–1.9)	0.01

## Data Availability

The data presented in this study are available on request from the corresponding author due to privacy and legal reasons.
